# Editorial: The relationship between diabetes and cancers and its underlying mechanisms

**DOI:** 10.3389/fendo.2022.992569

**Published:** 2022-08-09

**Authors:** Qiang Huo, Jing Wang, Nannan Zhang, Long Xie, Heshan Yu, Tao Li

**Affiliations:** ^1^ Clinical Research Center, Nanjing Jiangbei Hospital, Nanjing, China; ^2^ Center for Translational Medicine, Zibo Central Hospital, Zibo, China; ^3^ Blood Purification Center, Zibo Central Hospital, Zibo, China; ^4^ Department of Otolaryngology, Zibo Central Hospital, Zibo, China; ^5^ Department of Oncology, Shibo High-Tech Hospital, Zibo, China; ^6^ Department of Breast and Thyroid Surgery, Zibo Central Hospital, Zibo, China

**Keywords:** diabetes, antidiabetic drug, glucose metabolic disorder, cancer development, cancer risk, thyroid

Diabetes mellitus (DM) is one of the major life-threatening diseases resulting in increased health care costs, deteriorated quality of life, and premature death, as well as malignant neoplasm (1, 2). The prevalence of diabetes and malignancy has been increasing worldwide, presenting the hypothesis that there might be a potential direct relationship between DM and cancer morbidity or mortality. The association of DM with malignancy has been discussed for decades (3). Throughout the literature, about 8% to 18% of individuals suffering from cancer also present diabetes (4). Comprehensive studies have indicated increased cancer incidence and cancer death in both type 1 diabetes mellitus (T1DM) and type 2 diabetes mellitus (T2DM) worldwide (5–7).

The mechanisms underlying the relationship between DM and cancer have been investigated preliminarily (6, 7). Proliferation and apoptosis pathways may be involved. Factors including hyperglycemia, hyperinsulinemia, insulin-like growth factor 1 (IGF-1), oxidative stress, and sex hormones, were presented. Supraphysiological concentrations of insulin and/or glycemia to which the human tissues are exposed have also been discussed. The association could come from overlapping risk factors between DM and cancer, including aging, obesity, smoking, and physical inactivity. Physiology and treatments of DM may also lead to this association. On the other hand, cancer patients with DM were usually treated *via* less aggressive approaches and got worse prognoses, compared with those without diabetes, suggesting a better control (prevention, detection, and management) of the growing epidemic of DM in order to decrease the health care burden and to improve the quality of life (4). It is also reported that stringent management of hyperglycemia and insulin resistance in DM patients accompanying cancer could improve their overall survival. If causal, these associations may have great importance for public health considering the substantial medical burden of these diseases around the globe (4, 7, 8).

While the results of studies focused on the relationship between diabetes and cancer-specific incidence and/or death are inconsistent (9, 10). Both observational and experimental studies have suggested that medications used in DM treatment may be associated with either increased or decreased cancer risks. Meanwhile, selective reporting biases would also cause either false significance or an inflated estimation of the association. Thus, we should be careful when interpreting the study results, and methodological pitfalls should be avoided.

The aims and objectives of our Research Topic are to advance current knowledge on the clinical or biological mechanisms underlying the association of DM with cancer development.

During collecting, we reviewed many excellent works, in a variety of study protocols or article types. Hsu et al. conducted a state-level retrospective cohort study and demonstrated that, the use of an oral antidiabetic drug, dipeptidyl peptidase 4 (DPP-4) inhibitors, was associated with a decreased risk of hepatocellular carcinoma (HCC) in patients with type 2 DM and chronic hepatitis C virus (HCV) infection. Though this study is a retrospective observation, not randomized controlled trial (RCT) or prospective study, confounding factors including covariates and comorbidities were well controlled by the authors using multivariate Cox proportional hazard regression analysis.

Zhu and Qu briefly but comprehensively summarized previous studies and updated available evidence of the associations between different types of DM and several cancers. They also discussed the possible underlying biological mechanisms, and presented that the genetic factors, obesity, inflammation, oxidative stress, hyperglycemia, hyperinsulinemia, cancer therapies, and hypoglycemic drugs may play roles in the crosstalk between DM and malignancies.

Diabetic ketoacidosis (DKA) is characterized by hyperglycemia, ketosis and metabolic acidosis, which presents as a serious complication of certain disordered metabolic state (11). Thyroid storm (TS) is a pernicious endocrine emergency which could result in multiple organ dysfunction. Lino and colleagues presented us a rare case with simultaneous presentation of TS and DKA, vividly indicating that glucose metabolism and thyroid function are closely related, and normal thyroid function is essential for maintaining equilibrated glucose metabolism.

Then we go deeper. Now that the glucometabolic state and thyroid function go hand in hand, what about the correlation between glucose metabolism and thyroid cancer development? Zhao et al. conducted a hospital-based observational study to investigate the relationship between abnormal glucose metabolism parameters and differentiated thyroid carcinoma (DTC) development. The authors collected DTC patients with complete surgical pathological details and tested the glucose metabolism indicators before iodine-131 treatment. The result of their study suggested that abnormal glucose metabolism (DM, hyperinsulinemia, or insulin resistance) was associated with the development of DTC.

On the other hand, relationship between glucose metabolism and cancer recurrence should be noticed too. The 21-gene recurrence risk score (RS) is determined based on the expression levels of 16 cancer-related genes and 5 endogenous reference genes, and has been used to evaluate the risk of recurrence and expected benefit of adjuvant chemotherapy in females with estrogen receptor (ER)-positive, human epidermal growth factor-2 (HER2)-negative early-stage breast cancer (12). Zhu et al. evaluated the association of the 21-gene recurrence RS with metabolic profiles on breast cancer recurrence in hormone receptor (HR)-positive, HER2-negative early-stage breast cancers. In their large prospective study, the 21-gene RS was related to lower levels of body mass index (BMI), insulin, C-peptide, and to the absence of obesity, insulin resistance, hyperglycemia, and other favorable metabolic profiles. These findings also suggested that the prognostic impact of the 21-gene recurrence RS on breast cancer recurrence may present among female patients with favorable metabolic profiles.

Based on the current knowledge and studies collected in our Research Topic, the association between diabetes mellitus and cancers is very complicated, clinically and biologically. Several cancers or cancer therapies were associated with increased risk of diabetes mellitus. Meanwhile, the development of many types of cancers is more aggressive in patients with diabetes mellitus. Cancer screening in patients with DM, or diabetes screening in cancer patients should be conducted, as well as precautions or preventive intervention among these patients. Further study is warranted to investigate and confirm the relationship between diabetes and cancers.

## Author contributions

All authors have met the requirements for authorship. QH, JW and NZ were responsible for preparing the first draft and reviewing the topic materials. QH summarized and edited the manuscript. LX, HY and TL supervised the project. All authors have read and approved the final manuscript.

## Funding

QH is funded by the Science and Technology Development Project in Medicine and Health of Shandong Province (CN) (No. 2019WS306).

## Acknowledgments

We thank Yining Shen (Jining Medical University, China) for her assistance in collecting and collating information for this Research Topic.

## Conflict of interest

The authors declare that the research was conducted in the absence of any commercial or financial relationships that could be construed as a potential conflict of interest.

## Publisher’s note

All claims expressed in this article are solely those of the authors and do not necessarily represent those of their affiliated organizations, or those of the publisher, the editors and the reviewers. Any product that may be evaluated in this article, or claim that may be made by its manufacturer, is not guaranteed or endorsed by the publisher.
